# Elucidating the Neuroprotective Mechanisms of G‐3702 in Ischemic Stroke via Integrated Metabolomics and Computational Approaches

**DOI:** 10.1111/cns.70352

**Published:** 2025-04-23

**Authors:** Cong Wang, Fang Zhang, Qi Zheng, Junsong Wang

**Affiliations:** ^1^ Center for Molecular Metabolism, School of Environmental and Biological Engineering Nanjing University of Science and Technology Nanjing People's Republic of China; ^2^ Key Laboratory of Metabolic Engineering and Biosynthesis Technology Ministry of Industry and Information Technology Nanjing People's Republic of China; ^3^ School of Food Engineering Anhui Science and Technology University Fengyang People's Republic of China

**Keywords:** angiogenesis, Avb3 integrin pathway, G‐3702, ischemic stroke, machine learning, metabolomics, neuroprotection, pathway analysis

## Abstract

**Aims:**

Ischemic stroke (IS) remains a leading cause of disability worldwide, necessitating the development of more effective treatments. While DL‐3‐n‐butylphthalide (NBP) has shown promise in treating IS, its clinical application is limited by hepatotoxicity. G‐3702, a structural analog of NBP, has emerged as a potential alternative with reduced hepatotoxicity and proposed pro‐angiogenic effects. However, the precise mechanisms underlying G‐3702's therapeutic effects in IS remain unclear, hindering its optimization and the identification of novel therapeutic targets. This gap in understanding is particularly significant given the potential of pro‐angiogenic treatments to address ischemia‐induced vascular damage and improve long‐term recovery.

**Methods:**

Here, we employed an integrated approach combining metabolomics, transcriptomics, and machine learning to elucidate G‐3702's mechanisms of action in a photothrombotic stroke mouse model. Untargeted metabolomics and pathway analysis explored G‐3702's metabolic impacts, while network pharmacology and machine learning algorithms refined key therapeutic target identification. We validated computational insights through immunofluorescence and qPCR experiments.

**Results:**

Our results demonstrated that G‐3702 significantly improved neurological outcomes and reduced cerebral cortex necrosis in IS mice. Metabolomics implicated the Avb3 integrin pathway in G‐3702's pro‐angiogenic effects, while computational analyses highlighted the PI3K‐Akt and HIF‐1α pathways as central to this action. Machine learning algorithms prioritized potential biomarkers and targets, including BDNF, FGF2, ITGAV, ITGB3, SRC, and RHOA. Immunofluorescence confirmed enhanced angiogenesis, and qPCR demonstrated increased expression of these angiogenesis‐related genes following G‐3702 treatment.

**Conclusion:**

These findings suggest that G‐3702 promotes angiogenesis in the ischemic brain area primarily via the Avb3 integrin pathway, offering a mechanistic explanation for its therapeutic effects in IS. By elucidating G‐3702's mode of action, this study not only enhances its clinical potential but also contributes to the broader field of stroke treatment by identifying novel therapeutic targets. Our integrated approach to mechanism elucidation advances the understanding of pro‐angiogenic treatments for stroke and may serve as a model for future drug development efforts in IS and other complex neurological disorders. Ultimately, this work enhances G‐3702's potential for clinical translation as an improved stroke therapy and opens new avenues for optimizing post‐stroke recovery.

## Graphical abstract

G‐3702, a structural analog of NBP, improves neurological outcomes in ischemic stroke mice by promoting angiogenesis primarily through the Avb3 integrin pathway. Integrated metabolomics, transcriptomics, and machine learning analyses reveal key therapeutic targets and biomarkers, advancing our understanding of proangiogenic treatments for stroke and enhancing G‐3702's potential for clinical translation.

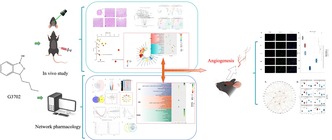

## 1 Introduction

Ischemic stroke (IS) stands as a principal cause of mortality and long‐term disability [1, 2, 3], presenting a significant challenge to healthcare systems worldwide. The condition occurs when there is an abrupt interruption in cerebral blood flow, leading to tissue ischemia and initiating a series of adverse events that can result in extensive neuronal damage. Despite technological advances in the establishment of acute stroke management protocols, effective treatment of IS is still fraught with difficulties [4, 5]. The multifactorial nature of IS, influenced by a complex interplay of genetic, environmental, and lifestyle factors, underscores the need for innovative diagnostic and therapeutic approaches.

Metabolomics has emerged as a powerful tool within systems biology [6], offering a comprehensive analysis of small molecule metabolites in biological samples. This approach has the potential to uncover disease‐specific biomarkers [7, 8] and to shed light on the molecular mechanisms implicated in the onset and progression of IS. Network pharmacology complements metabolomics by mapping the intricate web of biological interactions [9, 10], thereby predicting the impact of therapeutic compounds on disease pathways. Machine learning algorithms [10, 11], such as LASSO logistic regression, random forest, and SVM‐RFE, further refine the identification of key molecular targets, enhancing the precision of biomarker discovery and therapeutic interventions.

DL‐3‐n‐butylphthalide (NBP), a compound derived from Celery ( *Apium graveolens* L.), has been approved for IS treatment in China [12]. NBP is known for its neuroprotective properties [13, 14, 15, 16, 17], including antioxidative, antiapoptotic, and anti‐inflammatory effects, as well as its ability to enhance cerebral blood flow. NBP not only enhances the expression of angiogenic growth factors but also actively promotes angiogenesis in models of stroke. The primary focus of NBP is on elucidating the mechanisms underlying angiogenesis [18, 19, 20]. However, its clinical application is limited by moderate hepatotoxicity [21, 22]. To overcome this limitation, we synthesized a structural analogue of NBP, G‐3702, by replacing the carbonyl group of NBP with a boron hydroxyl group, which can significantly reduce the possibility of liver damage caused by the drug. Our previous study demonstrated the anti‐ischemic stroke efficacy of G‐3702, but the primary mechanism of G‐3702 is not yet clear [23].

In this study, we aimed to validate the primary mechanisms of G‐3702 treatment in IS (ischemic stroke) using a combination of metabolomics and computational biology approaches. Metabolomics offers a comprehensive analysis of small molecule metabolites in biological samples, which can uncover disease‐specific biomarkers. However, linking changes in metabolites to their upstream genes and proteins is a crucial step in understanding the underlying mechanisms.

To address this challenge, we integrated metabolomic data with upstream pathway analysis and machine learning of relevant disease transcriptomics datasets. By mapping how metabolic changes correspond to transcriptional variation, we aimed to gain insights into the molecular drivers of disease onset and progression. Additionally, network pharmacology helped us map the biological interactions implicated in the process. Machine learning algorithms such as LASSO logistic regression, random forest, and SVM‐RFE were employed to refine the identification of key molecular targets and biomarkers.

G‐3702 served as an illustrative example for this integrated approach. By applying the metabolomics–transcriptomics framework, we aimed to provide a more comprehensive theoretical basis for the clinical application of G‐3702 in IS. This methodology has the potential to offer a more holistic understanding of disease pathogenesis at the interface of metabolism and gene regulation, thus guiding future studies toward promising therapeutic targets and interventions.

## 2 Method

### 2.1 Animal Models and Experimental Procedures

Adult male C57BL/6J mice weighing 24–26 g were housed in a controlled environment with a constant temperature (25°C ± 1°C) and humidity (50% ± 10%). The mice were maintained on a 12‐h light–dark cycle and provided with ad libitum access to food and water. All experimental procedures were conducted in accordance with the guidelines and regulations approved by the Institutional Animal Care and Use Committee at Nanjing University of Science and Technology (Approval ID: ACUC‐NUST‐20231105). All mice were purchased from Nanjing Qinglongshan Animal Breeding Farm (Nanjing, China). The animal's body temperature was maintained at 37°C ± 0.5°C throughout the experiments using a controlled heating plate. All efforts were made to minimize both the number of animals used and any potential pain or distress experienced by the mice.

The mice were subjected to 4% isoflurane inhalation for induction and 1.5%–2.0% isoflurane for the maintenance of anesthesia. The scalp was incised to expose the skull surface and cleaned to reveal bregma and the target area for irradiation. Cerebral infarction was induced by activation of photosensitive Rose Bengal dye (4 mg/kg, Sigma‐Aldrich, St. Louis, MO, USA) in 0.9% NaCl solution. Two minutes prior to laser irradiation, Rose Bengal was injected through the abdominal cavity. The skull was illuminated for 15 min (wavelength 520 nm; 100 mW, Xian Leixinyanxiang). Sham‐operated rats received normal saline instead of Rose Bengal solution. After laser irradiation, the scalps were sutured, and the animals were allowed to recover in their home cages.

**FIGURE 1:**
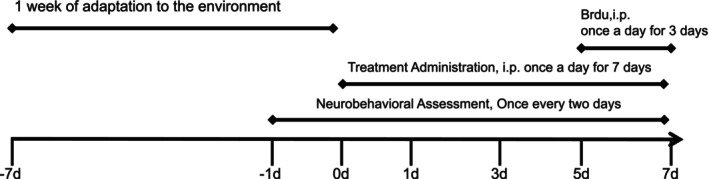
Experimental timeline for stroke model study.

All mice were randomly assigned into five groups: (1) negative control group (NC, *n* = 8), (2) stroke model group (M, *n* = 8), (3) G‐3702‐low dose treated (G‐3702 10 mg/kg/d) group (LD, *n* = 8), (4) G‐3702‐middle dose‐treated (G‐3702 20 mg/kg/d) group (MD, *n* = 8), (5) G‐3702‐high dose treated (G‐3702 40 mg/kg/d) group (HD, *n* = 8). The mice in the control group and model groups were i.g. administered olive oil (5 mL/kg) for 7 days, and the mean time the mice in G‐3702 treated groups were i.g. administered G‐3702 dissolved in olive oil (5 mL/kg) respectively. The brain was removed from the skull 1 h after the last administration (Figure 1).

### 2.2 Neurobehavioral Deficit Evaluation

To evaluate changes in neurological function associated with ischemia, the rats were subjected to a variety of somatosensory and motor tests before and after surgery. All testing was performed at the same time each day to minimize variability resulting from circadian rhythms.

The cylinder experiment is designed according to the characteristics of the animal's vertical attachment to the wall and upward exploration. To evaluate the degree of dependence of the mice on a certain side of the limb, mice were placed in a Plexiglas cylinder (15 cm in height with a diameter of 10 cm) and video‐recorded for 5 min to determine forelimb symmetry in exploratory rearing. The video footage was analyzed by calculating the time (in seconds) that each forelimb or both forelimbs were placed on the cylinder wall. The asymmetry index is as follows: (% ipsilateral use) – (% contralateral use). The video was analyzed by individuals who were blinded to these experiments.

The grid‐walking task was carried out as previously described with minor modifications [24, 25, 26]. The device is a 32 cm × 20 cm × 50 cm (length × width × height) iron frame with a 12‐mm‐square wire mesh above it. A mouse was placed individually on the wire grid for 3 min while being video‐recorded. Foot faults and the total normal steps (number of foot faults + number of nonfaults) of the forelimb were counted. The foot fault index was calculated as the number of foot faults/total steps × 100%. A camera was positioned beneath the device to capture video footage to assess the stepping errors (foot faults). A step was considered a foot fault if the forelimbs passed through the grid hole, or the mouse was resting with the grid at the level of the wrist.

### 2.3 Histopathology Examination

The brain tissue was fixed in 4% paraformaldehyde overnight, then embedded in paraffin. A series of 5‐μm‐thick sections was cut from the treated brain tissues, finally stained with hematoxylin and eosin stain (HE) for histopathological observation.

### 2.4 Metabolomic Profiling of Ischemic Brain Tissues Using LC–MS and Bioinformatics Analysis

#### 2.4.1 Sample Preparation and LC–MS Detection Methods for Brain Tissue Metabolomics

For the analysis of metabolomics, a detailed procedure encompassing sample preparation, LC–MS analysis, and data processing was outlined. Initially, a 100 μL brain tissue sample was mixed with 400 μL of pre‐cooled methanol, vortexed, frozen, and then centrifuged to precipitate proteins. The supernatant was collected, and the pellet was processed further for LC–MS analysis. Quality control (QC) samples were also prepared to ensure the mass spectrometry's stability and repeatability.

The LC–MS analysis was conducted using a Triple TOF 5600+ system with an electrospray ionization source. A gradient elution method was employed using formic acid and methanol as mobile phases. The analysis captured full‐scan mass spectra in the negative ion mode, with specific settings for voltage, declustering potential, collision energy, and gas pressures. Automated MS/MS scans were performed for the most intense ions to ensure accuracy and calibration.

Data processing involved converting raw files to the mzML format using ProteoWizard software, followed by ion feature extraction with XCMS software. Metabolite identities were assigned by matching spectra to the HMDB spectral library. The data were normalized using R, and the mixOmics R package was utilized for further analysis. Potential biomarkers were identified based on VIP scores and *t*‐tests.

#### 2.4.2 Pathway Analysis of Metabolites and Associated Genes and Proteins

Metabolite Set Enrichment Analysis (MSEA) was made using MetaboAnalyst 5.0 (https://www.metaboanalyst.ca/) on the KEGG database (http://www.kegg.jp/) and MetScape in Cytoscape. Based on the gene and protein interaction data in the Pathway Commons database (http://www.pathwaycommons.org/), pathway analysis was performed on the upstream genes and proteins associated with differentially expressed metabolites across HD, MD, and LD groups, using the R package CePa. Specifically, CePa was used to analyze the gene/protein interactions involved in metabolic pathways of differentially expressed metabolites among the three groups. Pathways common and significant to all groups were identified to elucidate core metabolic pathways influenced by G‐3702 treatment. This analysis aimed to explore the regulatory networks between metabolites and their associated genes/proteins modulated by G‐3702.

### 2.5 Bioinformatics Approaches

#### 2.5.1 Identification of Stroke‐Related Genes From GEO Dataset

Differentially expressed genes (DEGs) related to stroke were identified based on the GEO dataset GSE58294 [27]. Using the Limma package in R software, we conducted differential expression analysis on standardized chip data with criteria such as |logFC| > 0.5 (where logFC represents log fold‐change) and a *p* value < 0.01. DEGs were visualized as a volcano plot and clustered heatmap using the R package ggplot2 and pheatmap.

#### 2.5.2 Target Prediction of G‐3702 and Construction of Protein–Protein Interaction Network

The potential targets of G‐3702 were predicted based on its structure using three online tools: SwissTargetPrediction (http://old.swisstargetprediction.ch/), super‐pred (https://prediction.charite.de/subpages/targetprediction.php), and sea (http://218.8.241.248:8080/SEA3/index.html). The predicted targets were then integrated with differentially expressed genes (DEGs) from dataset GSE58294 to identify overlapping targets as candidate genes. Protein–protein interaction (PPI) data was downloaded from the STRING database (https://cn.string‐db.org/) to construct the PPI network. The top 10 hub genes in the network were visualized using Cytoscape.

#### 2.5.3 Integrated Bioinformatic Analysis of Key Targets of G‐3702 and Pathways

To pinpoint key genes modulated by G‐3702, we applied three machine‐learning algorithms to the 22 candidate genes predicted from target prediction and analysis of the GSE58294 dataset. The algorithms used were LASSO logistic regression, random forest, and SVM‐RFE via their corresponding R packages. The algorithms helped analyze potential molecular targets of G‐3702 related to IS. The intersections of genes prioritized by the three algorithms were identified using Venn diagrams generated with the “VennDiagram” R package.

Functional enrichment analysis of Gene Ontology (GO) terms and Kyoto Encyclopedia of Genes and Genomes (KEGG) pathways was performed to gain mechanistic insights into the shortlisted genes. The “clusterProfiler” and “tinyarray” R packages were used for systematically investigating the functions and roles of the common genes in governing stroke pathogenesis at the molecular level. This analysis aimed to elucidate how modulation of these target genes by G‐3702 may exert therapeutic effects in IS.

### 2.6 Experimental Verification

#### 2.6.1 Immunohistochemistry (IHC)

Frozen brain tissues were sliced into 10 μm sections and prepared for immunostaining as described previously. The brain sections were washed with PBS (3 × 10 min) and blocked with blocking solution (10% goat serum containing 0.1% Triton X–100) at room temperature for 1 h. The sections were then incubated with the primary antibodies against BrdU (Mouse 1:300), CD31 (Rabbit 1:100) in blocking solution at 4°C overnight. Subsequently, the sections were rinsed three times with PBS and then incubated with fluorescence‐labeled secondary antibodies (1:200, Proteintech, China) at room temperature in the dark for 1 h. The sections were washed again and stained with DAPI to label the nucleus. Finally, the sections were cover slipped with 50% glycerine and photographed under a fluorescence microscope (DFC310, Leica, Germany). Six views of each group were selected randomly and used for the following analysis. Each experiment was performed three times.

#### 2.6.2 Quantitative Real‐Time PCR (qPCR)

Total RNA was extracted from the tissue samples of each experimental group, and 5 μg of total RNA was reverse transcribed into cDNA using a reverse transcription kit (Vazyme) according to the manufacturer's instructions. qPCR was performed in a 25 μL reaction system, which included SYBR Green/Fluorescein qPCR Master Mix, forward and reverse primers, and the synthesized cDNA. The ABI7500 real‐time PCR system (Applied Biosystems) was utilized for the PCR amplification. The relative mRNA expression levels in the thoracic aorta tissues were determined using the 2^−ΔΔCt^ method, with GAPDH serving as the endogenous control for normalization. The specific PCR primers employed in this study are listed in Table 1.

**TABLE 1:** Sequences of the primers in this study.

Gene	Sequences (F)	Sequences (R)
GAPDH	CATCACTGCCACCCAGAAGACTG	ATGCCAGTGAGCTTCCCGTTCAG
BDNF	CATCCGAGGACAAGGTGGCTTG	GCCGAACTTTCTGGTCCTCATC
FGF2	AAGCGGCTCTACTGCAAGAACG	CCTTGATAGACACAACTCCTCTC
ITGAV	AGGAGAAGGTGCCTACGAAGCT	GCACAGGAAAGTCTTGCTAAGGC
ITGB3	CATGGATTCCAGCAATGTCCTCC	TTGAGGCAGGTGGCATTGAAGG
NAMPT	GGCACCACTAATCATCAGACCTG	AAGGTGGCAGCAACTTGTAGCC
SRC	CTGCTTTGGCGAGGTGTGGATG	CCACAGCATACAACTGCACCAG
RHOA	TCTGTCCCAACGTGCCCATCAT	CTGCCTTCTTCAGGTTTCACCG
VEGFA	TTGCCTTGCTGCTCTACCTCCA	GATGGCAGTAGCTGCGCTGATA

### 2.7 Statistical Analysis

R software (version 4.2.3) was used for all statistical analyses. Data were represented as mean ± SEM. Student's *t*‐test was used for comparisons between two groups. For multiple group comparisons, one‐way analysis of variance (ANOVA) was performed followed by Tukey's post hoc test to calculate *p* values and determine differences among groups.

## 3 Results and Discussion

### 3.1 Histopathological Examination and Behavioral Tests

**FIGURE 2:**
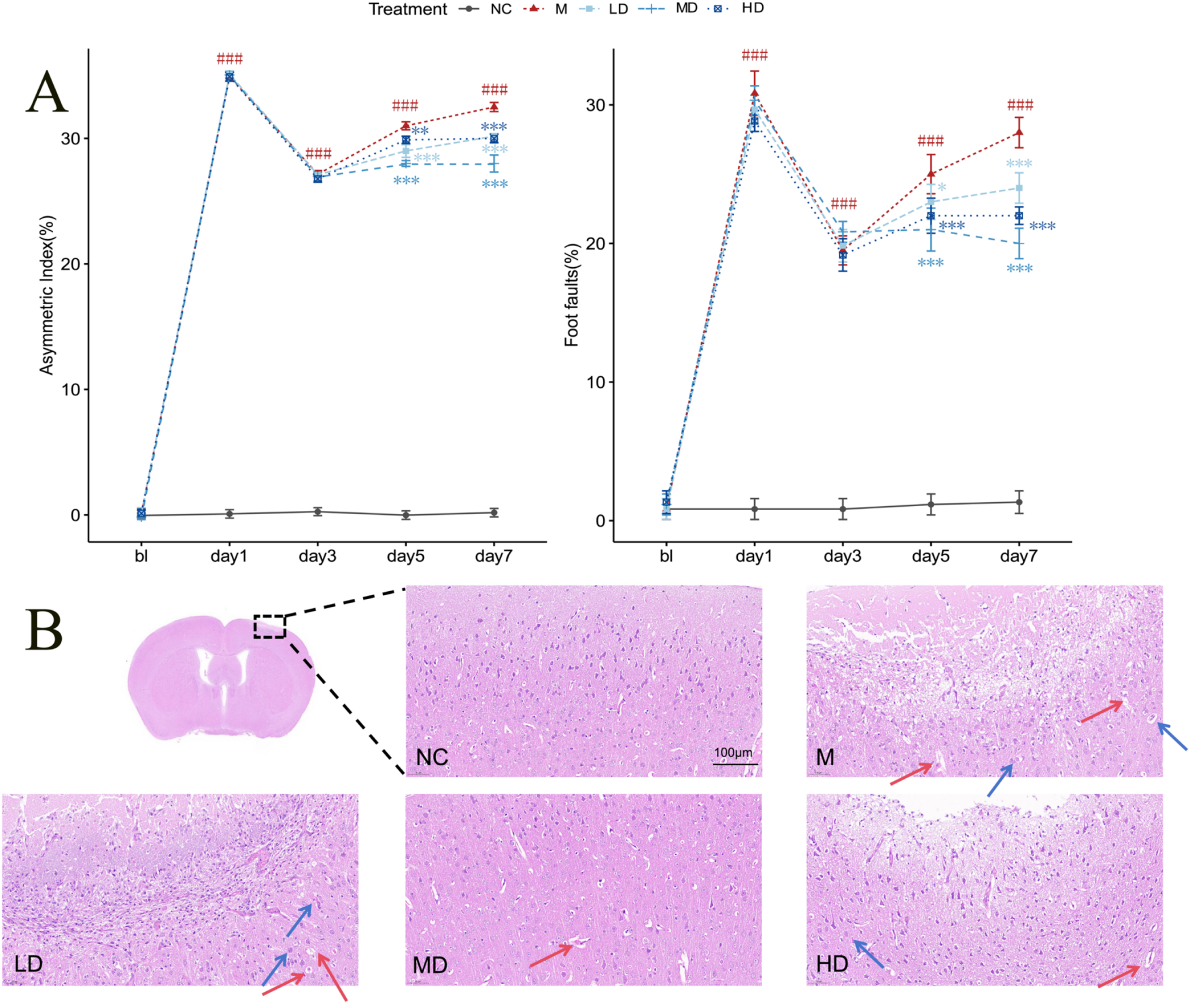
G‐3702 attenuated ischemic stroke‐induced behavioral deficits and neuropathological changes. (A) Behavioral performance was assessed by the cylinder and foot fault tests at Day 7 poststroke (mean ± SEM). ###*p* < 0.001 vs. CON; *p < 0.05, **p < 0.01, ***p < 0.001, vs. M group. (B) Representative microscopic images of brain sections in the ischemic penumbra stained with HE. Scale bar represents 100 μm. Red arrows point to pyknotic neurons and blue arrows indicate vacuole‐like structures. Group information: Negative control group (NC), stroke model group (M), G‐3702‐low dose‐treated group (LD), G‐3702‐Middle dose‐treated group (MD), G‐3702‐high dose‐treated group (HD).

The forelimb asymmetry index and percentage of left forelimb foot faults were used to assess behavioral performance after IS surgery. At 24 h postsurgery, motor function was severely impaired in all surgical groups. This confirmed successful induction of ischemic injury. While performance stabilized over the next few days in controls (Figure 2A), the model group displayed progressively worsening behavioral outcomes. By Day 7 poststroke, the forelimb asymmetry index revealed a prominent bias toward the right (ipsilateral) forelimb (*p* < 0.001) in the model group compared to normal controls, indicating motor impairment on the left side. Similarly, the percentage of left forelimb foot faults was significantly increased (*p* < 0.001) in the model group versus controls. However, G‐3702 treatment dose‐dependently improved behavioral performance, with the medium dose exhibiting the best effect and low dose the lowest efficacy. The high dose showed intermediate effectiveness. These results demonstrate that G‐3702 mitigated IS‐induced neurological deficits.

In the HE staining of brain sections (Figure 2B), prominent pathological changes were observed in the penumbra region of the ischemic hemisphere in the model group. Neurons in this region were significantly decreased compared with controls. Many neurons appeared swollen with loosened structure and deeply stained, indicative of pyknotic nuclei (red arrows). Boundaries between the neuron nuclei and cytoplasm were blurred; some nuclei were lost, forming vacuole‐like structures (blue arrows). These findings demonstrated neuronal damage in the ischemic penumbra. G‐3702 treatment significantly reduced the area of necrosis and liquefaction in the cerebral cortex of the injured hemisphere. Neuropathological changes were also markedly alleviated by G‐3702 treatment. The results showed that G‐3702 protected neurons from ischemic injury in the vulnerable penumbra region surrounding the ischemic core.

### 3.2 Effects of G‐3702 Treatment on the Brain Metabolism in Photothrombotic Stroke Mice

**FIGURE 3:**
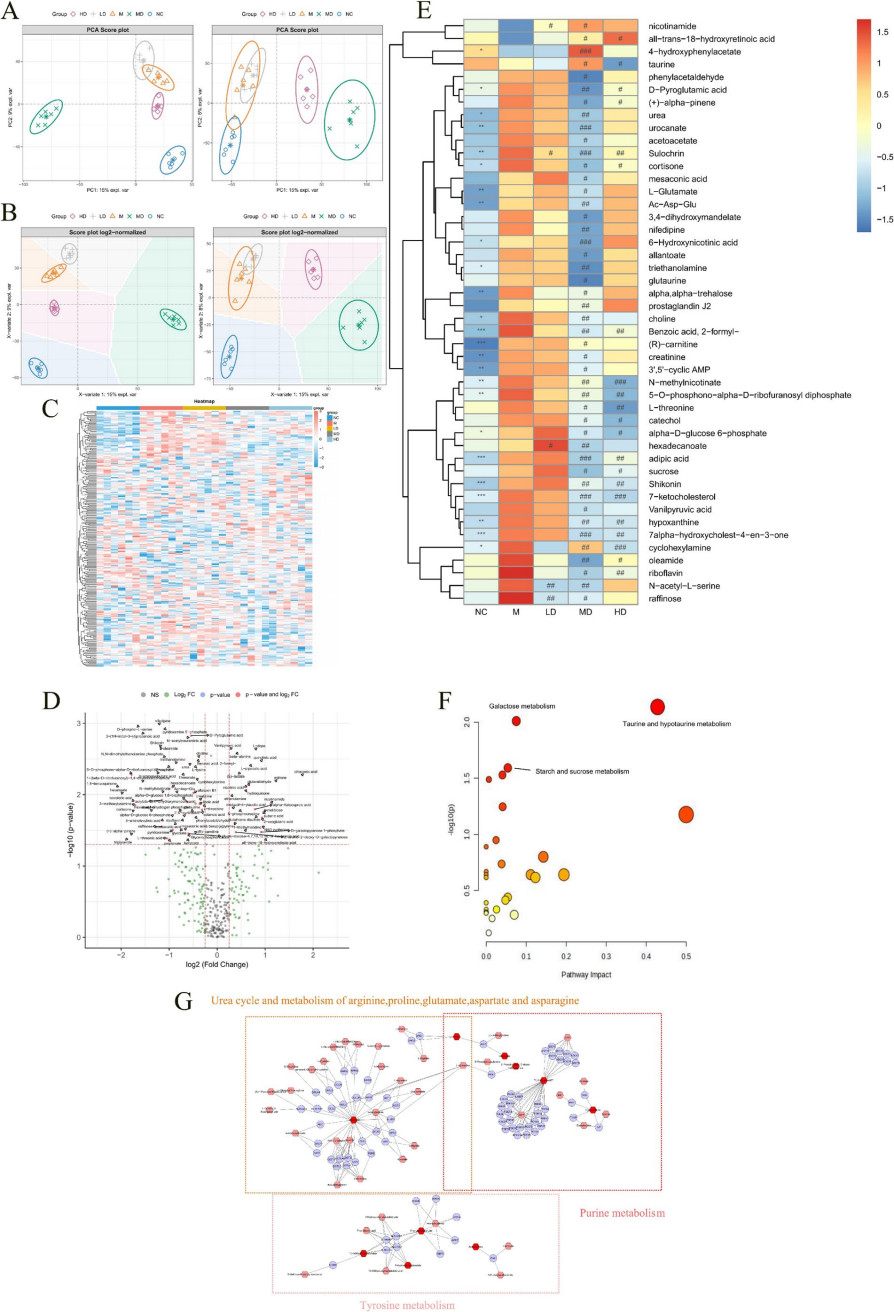
Analysis of total metabolites and screening and enrichment of differential metabolites. (A) PCA plot of brain metabolites between five groups in positive and negative ion modes. (B) PLS‐DA plot of brain metabolites between five groups in positive and negative ion modes. (C) Heatmap of all metabolites. (D) Volcano plot of different metabolites between MD and M. (E) Heatmap of significantly different metabolites between five groups. *p < 0.05, **p < 0.01, ***p < 0.001 M vs. CON; #*p* < 0.05, ##*p* < 0.01, ###*p* < 0.001 different treatment group vs. M. Enriched metabolic pathways of differential metabolites in MetaboAnalyst (F) and Metscape (G).

Untargeted metabolomic profiling was performed on stroke brain tissues to investigate the effects of G‐3702 treatment. Multivariate analysis including PCA [28] and PLS‐DA [24, 29] was used to analyze the metabolic profiles. PCA revealed distinct clustering patterns between the model (M) and normal control (NC) groups, reflecting stroke‐induced metabolic changes. The G‐3702 treatment groups also showed differential patterns compared with M and NC groups, indicating potential metabolic alterations after drug intervention (Figure 3A).

PLS‐DA further confirmed metabolic differences between groups. Specifically, M and NC groups were well separated, as were the G‐3702 dose groups (Figure 3B). These differences may be related to the dosage effects of the drug, indicating that metabolomics can be used to demonstrate the effects of drug intervention. In total, 271 common metabolites were identified across groups (Figure 3C). Further analysis focused on differences between the middle‐dose (MD) and M groups, resulting in 46 differential metabolites, including 25 reversed by MD treatment (Figure 3D,E).

This study provided novel insights into the multifaceted mechanisms by which G‐3702 exerts therapeutic effects in IS through modulating disrupted metabolic pathways (Figure 3F,G). As evidenced by elevated levels of glutamate and glycolytic/phosphorylated intermediates in the stroke model, oxidative stress and energy metabolic dysfunction were induced under ischemic conditions.

Heightened cerebral energy demand driven by hyperglycolysis and glutaminolysis caused neuronal excitotoxicity marked by glutamate elevation [30]. This occurs as impaired oxidative glucose metabolism prompts alternative substrate utilization like glutamate to fuel oxidative phosphorylation. Upregulation of this compensatory mechanism long‐term may become pathological. However, G‐3702 treatment, particularly at the optimal medium dose, effectively mitigated excitotoxicity by reducing glutamate to near‐normal levels.

Glycolytic upregulation in the model was indicated by increased raffinose, sucrose, glucose‐6‐phosphate, and trehalose, reflecting perturbed sugar metabolism [31]. The medium dose significantly attenuated these dysregulations, suggesting therapeutic restoration of disrupted carbohydrate processing in stroke through calibrated normalization of injury‐induced disturbances.

Modulation of the urea cycle [32], purine metabolism [33] and tyrosine metabolism [34] by G‐3702 also supported its protective effects on hepatic function, antioxidant capacity, neurotransmitter balance, and recovery mechanisms.

The elevation of urea levels in the stroke model reflects increased protein catabolism and hepatic stress. G‐3702's ability to lower urea may indicate alleviation of metabolic stress through urea cycle modulation.

Regarding purine metabolism, accumulations of allantoate and hypoxanthine in stroke are suggestive of accelerated ATP degradation and oxidative stress under hypoxic conditions, contributing to cellular damage. G‐3702's reduction of these metabolites points to its protective role against oxidative stress by regulating purine metabolism.

Additionally, increased 5‐O‐phosphono‐alpha‐D‐ribofuranosyl diphosphate (5‐PRPP) levels in stroke denote heightened demand for nucleotide synthesis during DNA repair processes [35]. The ability of G‐3702 to decrease 5‐PRPP reflects support for neuronal recovery mechanisms.

In tyrosine metabolism, elevated phenylacetaldehyde [36] in stroke may relate to exacerbated inflammatory processes resulting from disrupted aromatic amino acid catabolism. G‐3702's lowering of phenylacetaldehyde suggests its anti‐inflammatory effects through modulation of this pathway. The drug also influenced 3,4‐dihydroxymandelate and 4‐hydroxyphenylacetate, tying its effects to neurotransmitter and gut microbiota homeostasis—critical systems impacted in stroke.

Notably, G‐3702 elevated neuroprotective taurine levels while decreasing associated 5‐L‐glutamyl‐taurine, restoring disrupted taurine/glutathione homeostasis [37, 38]. This integrated metabolomics analysis provides novel insights into the mechanisms by which G‐3702 exerts multimodal neuroprotective effects against IS through coordinated modulation of metabolic pathways at the optimal medium dosage.

### 3.3 G‐3702 Modulates Stroke‐Induced Perturbations in Integrin Signaling Pathways

**FIGURE 4:**
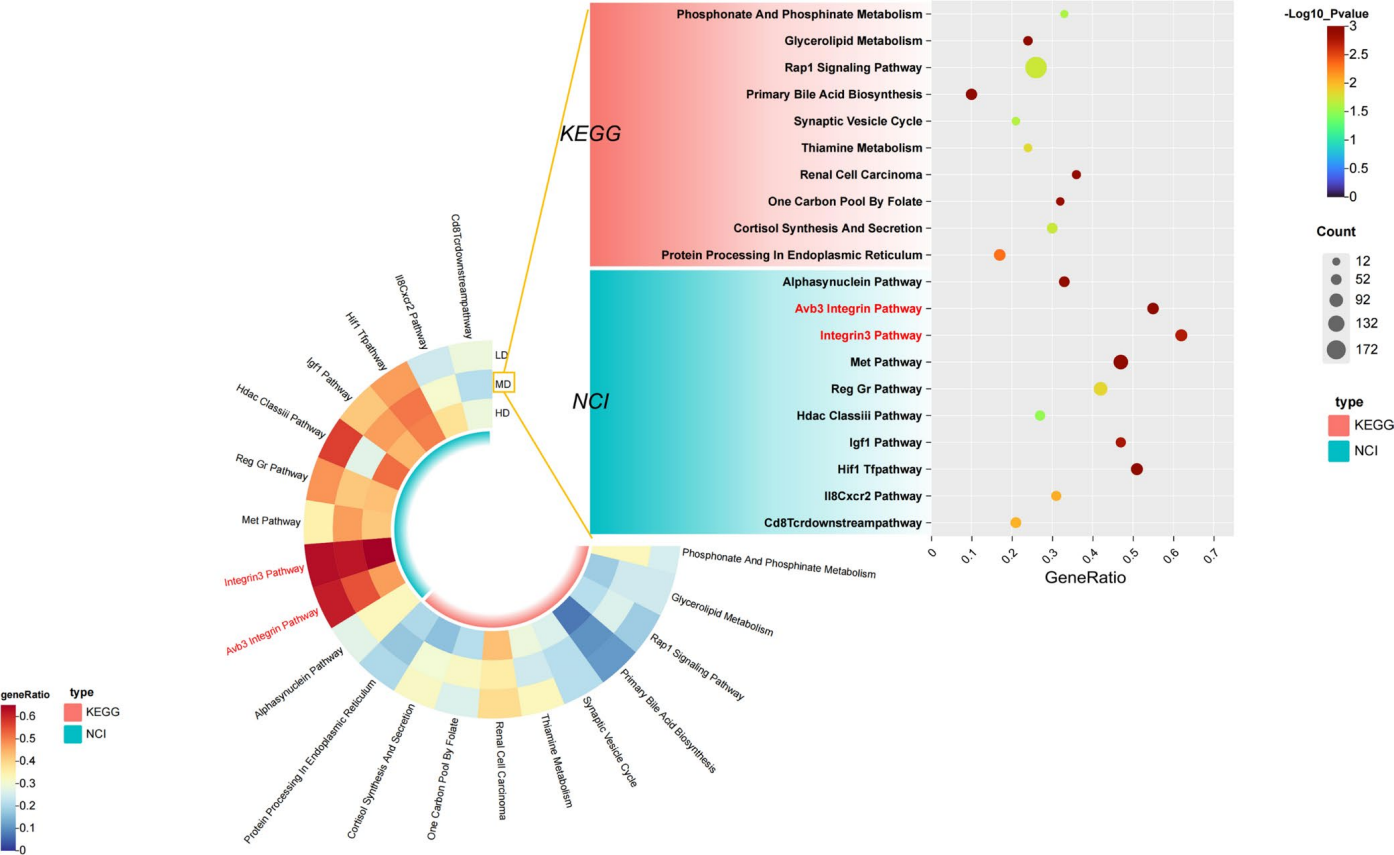
Common pathways screened among the three dosage groups of G‐3702. The heatmap displays the gene ratios of different pathways in the KEGG and NCI databases for different groups. The bubble plot displays the number of genes, gene ratios, and significance levels of the M group in the KEGG and NCI databases.

Pathway analysis of differential metabolites and associated genes/proteins identified core pathways enriched across dosage groups (Figure 4). The Avβ3 Integrin and Integrin3 pathways exhibited high gene ratios above 0.45, suggesting significant enrichment. Both pathways involve integrins, which are heterodimeric receptors mediating cell–cell and cell–matrix interactions critical to stroke outcomes [39].

In the acute phase after stroke, integrins may regulate blood flow by modulating vascular contraction/dilation via interactions with endothelial cells, thus influencing oxygen delivery. During later recovery, integrins play an important role in angiogenesis alongside VEGF [40]. Studies show active angiogenesis occurring 3–4 days after human brain ischemia [41], as vessel number significantly increased by Day 3.

Angiogenesis involves growth factors, receptors, and integrins coordinating endothelial cell survival, proliferation, migration, and new vessel formation—a process vital for tissue repair. VEGF signals through VEGFR‐2, differentially inducing its interaction with β3 integrin. Only αvβ3 integrin associates with VEGFR‐2 upon VEGF treatment, highlighting its key role downstream of VEGF in processes like tumor angiogenesis. Upregulation of αvβ3 integrin further implies its involvement.

Most well‐known angiogenic factors such as integrin αvβ3 increased within 1 h of injury [42]. The results demonstrate enrichment of pathways implicating integrins, especially αvβ3 [43], in acute blood flow regulation and longer‐term angiogenesis via VEGF signaling. This implies G‐3702's protective effects may involve modulating these processes through integrin pathways.

### 3.4 Transcriptome Profiling and Target Prediction Reveal Hub Genes Modulated by G‐3702 Following Stroke

**FIGURE 5:**
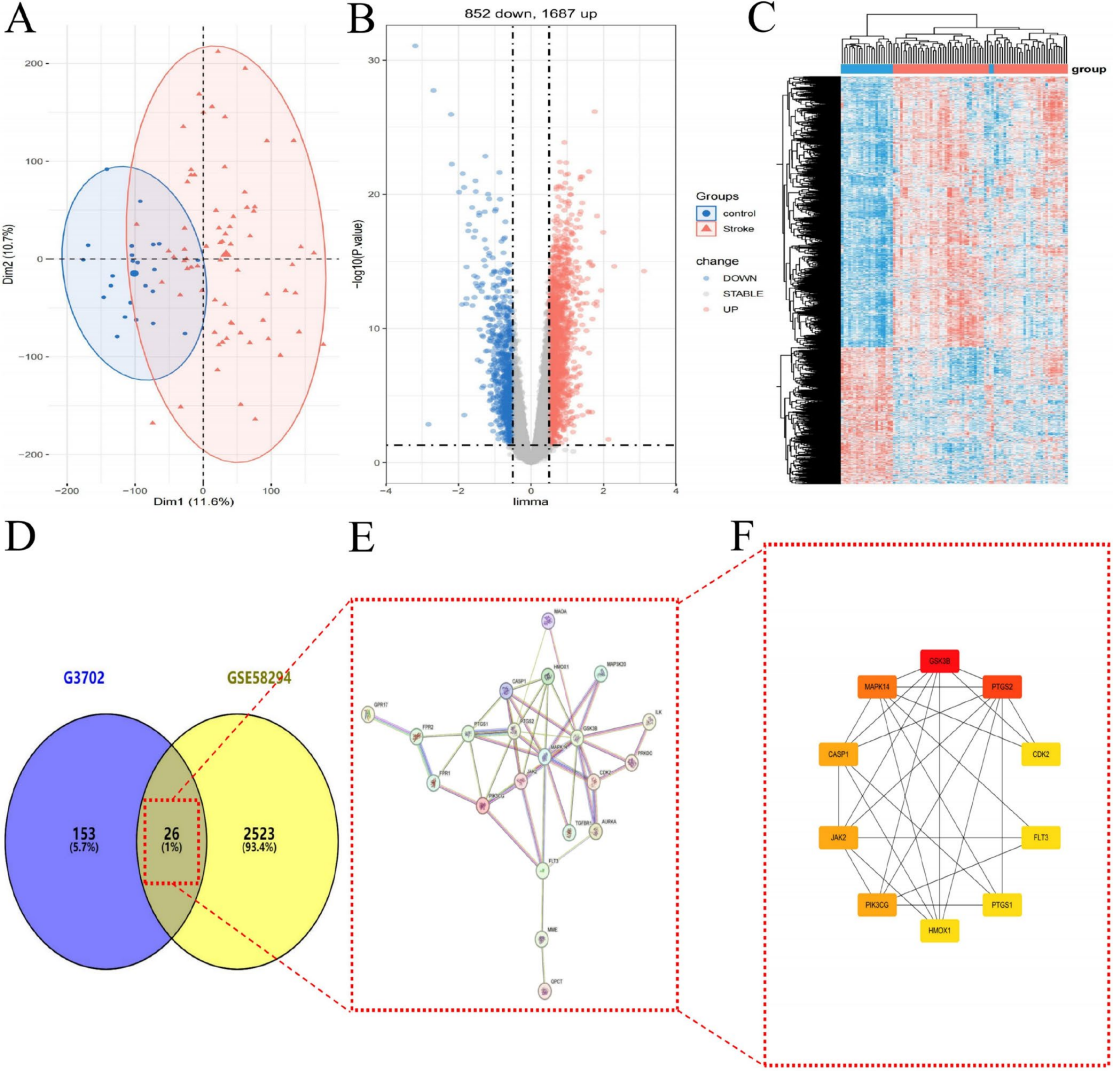
Bioinformatics analysis reveals the gene expression landscape modulated by G‐3702 following stroke. (A) PCA plot of all genes in the GSE58294 microarray dataset, showing separation of control (blue) and stroke (red) groups. (B) Volcano plot illustrates 2549 differentially expressed genes (DEGs) between control and stroke groups, identified using a cutoff of *p* < 0.05 and |logFC| > 0.5. (C) Heatmap displays expression levels of all identified DEGs. (D) Venn diagram shows 26 hub genes overlapping between targets predicted for G‐3702 through bioinformatics tools and DEGs from microarray analysis. (E) Protein–protein interaction network constructed using the STRING database, incorporating the 26 overlapping genes. (F) Core genes extracted from the network using degree algorithm, revealing candidate targets modulated by G‐3702 involved in the ischemia‐induced transcriptome changes.

PCA was performed on gene expression data from the GSE58294 microarray dataset. As shown in Figure 5A, the control (blue) and stroke (red) groups formed two distinct clusters in PCA space, explaining 22.3% of the variation.

Differential expression analysis identified DEGs between groups using a cutoff of *p* < 0.05 and |logFC| > 0.5. A total of 2549 DEGs were obtained, including 857 downregulated and 1692 upregulated genes (Figure 5B). The heatmap displays expression levels of all DEGs (Figure 5C).

Bioinformatics tools SwissTargetPrediction, super‐pred, and sea were used to predict 179 potential targets of G‐3702. Cross‐referencing these with the 2549 DEGs identified 26 common genes (Figure 5D). The STRING database was queried to construct a PPI network incorporating these 26 genes, retaining 22 connected nodes (Figure 5E).

Network analysis identified the top 10 hub genes (GSK3B, CASP1, JAK2, MAPK14, PIK3CG, PTGS2, PTGS1, CDK2, FLT3, HMOX1) with the highest degrees in the PPI network. The network was visualized using Cytoscape (Figure 5F), revealing candidate genes modulated by G‐3702 involved in the stroke transcriptome.

### 3.5 Consensus Targeting of Core Stroke‐Related Genes by Integrated Machine Learning Analyses

**FIGURE 6:**
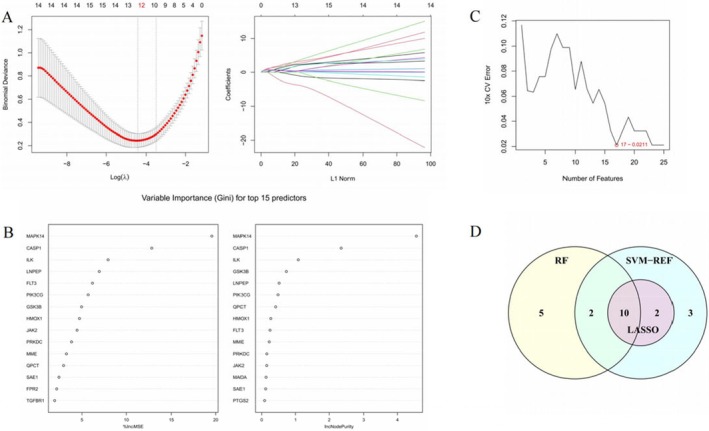
Ten hub genes screened by ML. (A) LASSO regression algorithm. (B) Two random forest algorithms. (C) SVM‐RFE algorithm. (D) The Venn diagram of 10 overlapped genes.

Building on the previous PPI network analysis, machine learning techniques were applied to further refine key genes modulated in stroke pathogenesis. LASSO regression identified 12 hub genes corresponding to the optimal regularization parameter for classifying control vs. stroke samples from the GSE58294 dataset (Figure 6A). Random forest analysis established the 17 most important features based on “%IncMSE” and “IncNodePurity” criteria (Figure 6B). SVM‐RFE concurred by recognizing the first 17 DEGs as yielding the lowest error and highest accuracy in classification (Figure 6C).

The specific genes identified by each algorithm are tabulated in Table 2. Their intersection yielded 10 overlapping genes prioritized across methods (Figure 6D): CASP1, FLT3, GSK3B, HMOX1, ILK, LNPEP, MAOA, MAPK14, PIK3CG, PTGS1, SAE1.

**TABLE 2:** Ten hub genes screened by machine learning.

RF	SVM‐REF	LASSO	COMMON GENES
MAPK14	MAPK14	AURKA	CASP1
CASP1	PTGS1	CASP1	FLT3
ILK	ILK	FLT3	GSK3B
LNPEP	HMOX1	GSK3B	HMOX1
FLT3	CDK2	HMOX1	ILK
PIK3CG	LNPEP	ILK	LNPEP
GSK3B	SAE1	LNPEP	MAOA
HMOX1	FLT3	MAOA	MAPK14
JAK2	PIK3CG	MAPK14	PIK3CG
PRKDC	CASP1	PIK3CG	SAE1
MME	GSK3B	PTGS1	
QPCT	GPR17	SAE1	
SAE1	MAOA		
FPR2	JAK2		
TGFBR1	AURKA		
MAOA	FPR1		
PTGS2	FPR2		

Functional analysis of these consensus targets was conducted to mechanistically contextualize their roles in stroke pathogenesis and how modulating their expression may underlie G‐3702's therapeutic effects, as revealed through earlier bioinformatics analysis. This complementary multi‐algorithm approach aimed to comprehensively uncover key molecular players in ischemic injury.

### 3.6 Integrated Pathway Analysis Implicates Conserved Pro‐Angiogenic Networks Modulated by G‐3702

**FIGURE 7:**
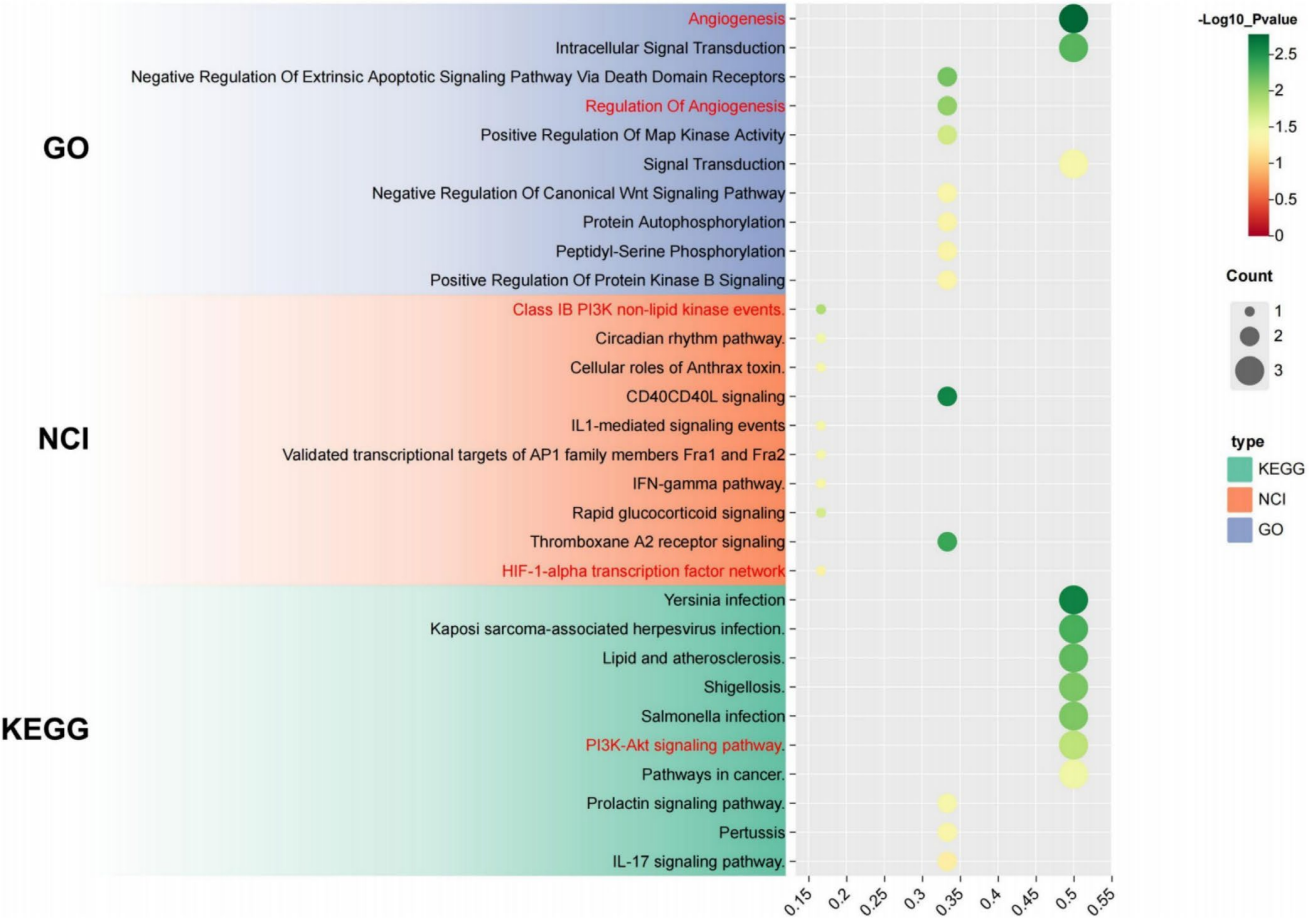
Functional enrichment and pathway analysis of the six common targets. GO enrichment analysis revealed significant association with angiogenesis‐related biological processes, including “Regulation of Angiogenesis” and “Angiogenesis.” Pathway analysis, utilizing both the NCI and KEGG, identified the PI3K‐Akt signaling pathway as a key pathway common to both databases. The PI3K‐Akt pathway, critical for processes such as endothelial cell survival, migration, and angiogenic factor expression, is implicated in promoting angiogenesis. Furthermore, specific events like Class IB PI3K nonlipid kinase signaling and the hypoxia‐inducible factor 1‐alpha (HIF‐1α) transcription network were identified, highlighting mechanisms through which these targets might regulate angiogenesis in response to hypoxia. These findings suggest that G‐3702 potentially promotes poststroke angiogenesis by modulating the PI3K‐Akt and HIF‐1α pathways.

The six common targets (MAPK14, CASP1, FLT3, PIK3CG, GSK3B, HMOX1) intersecting between the PPI network and machine learning analyses were subjected to functional enrichment and pathway analysis. GO analysis revealed enrichment of angiogenesis‐related terms like “Regulation of Angiogenesis” and “Angiogenesis,” indicative of their relevance to this process (Figure 7).

Pathway analysis utilizing the NCI‐PID and KEGG identified the PI3K‐Akt signaling pathway [44] as a major enriched pathway common to both databases. The PI3K‐Akt pathway plays essential roles in various processes, including angiogenesis. It influences the expression of angiogenic factors and facilitates cellular behaviors contributing to new vessel formation [45, 46, 47].

Specifically, the NCI database implicated Class IB PI3K nonlipid kinase events and HIF‐1α transcription factor network involvement. Class IB PI3K activation induces angiogenic factor expression and promotes endothelial cell survival/migration during angiogenesis. Under hypoxia [48, 49], HIF‐1α stabilizes to induce targets like VEGF that are central to the angiogenic response.

These analyses indicate G‐3702 may promote poststroke angiogenesis by modulating conserved pathways tied to the consensus targets identified through integrated bioinformatics and machine learning. Coordinated regulation of PI3K‐Akt signaling and HIF‐1α activity suggests mechanisms by which G‐3702 exerts neuroprotective effects.

### 3.7 Pro‐Angiogenic Effects of G‐3702 in Ischemic Stroke

Previous bioinformatics analyses implicated angiogenesis as a key process modulated by G‐3702 in stroke. To experimentally validate its proangiogenic effect, we assessed vascular parameters in brain sections immunofluorescently stained for CD31. The severity of poststroke injury can influence the extent of stimulated neurogenesis and angiogenesis. To determine whether enhanced repair was solely due to exacerbated damage, we evaluated proliferating endothelial cells by dual labeling of BrdU and CD31. CD31 serves as an indicator for microvessel density, with Cy3 rendering CD31 in red fluorescence. Cells in a proliferative state can be labeled with BrdU, which emits green fluorescence with FITC, while DAPI stains the cell nuclei blue.

**FIGURE 8:**
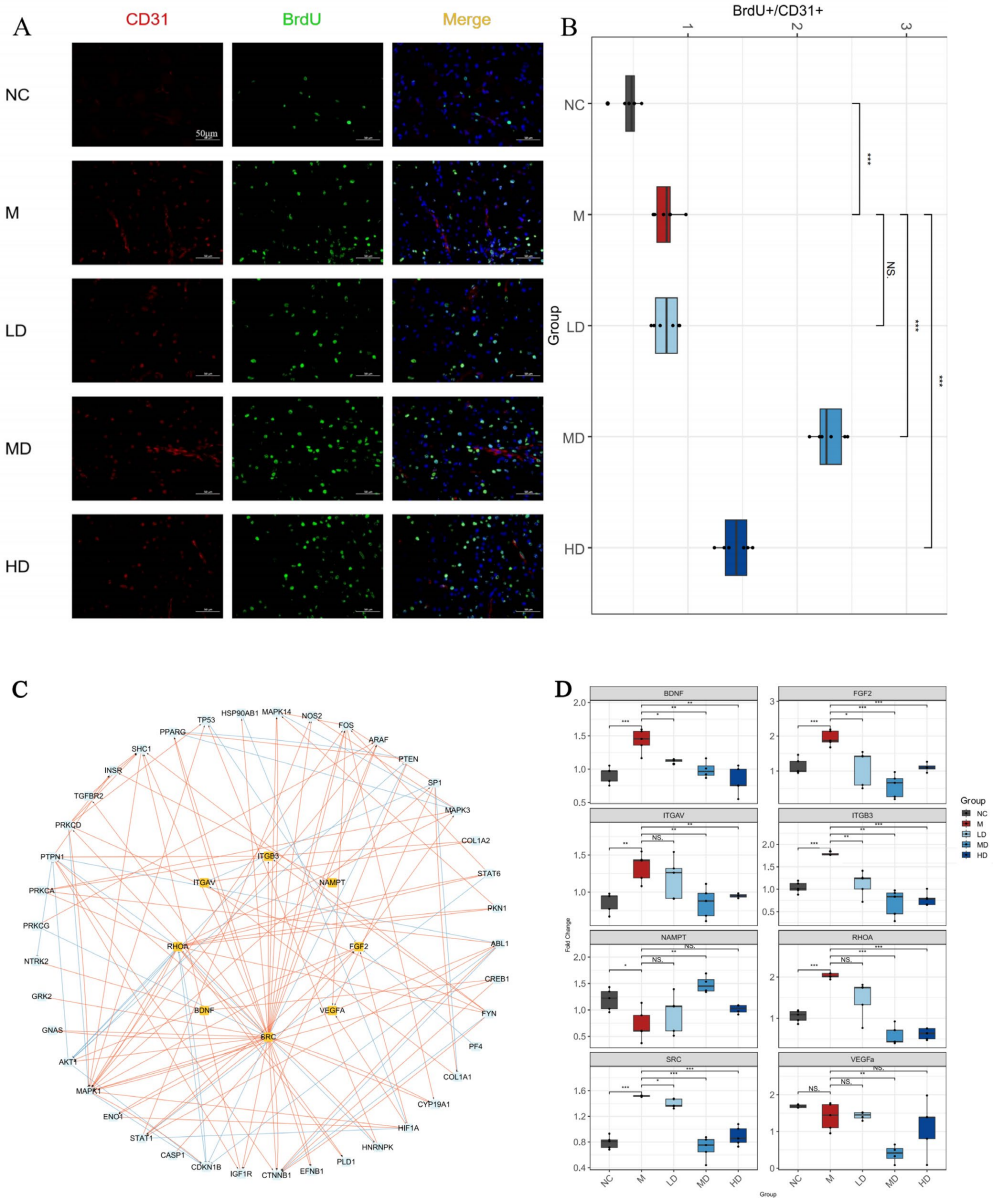
Immunofluorescence detection and qPCR validation of angiogenesis‐related markers in ischemic stroke. (A) Representative confocal fluorescence microscope images showing immunohistochemical labeling for BrdU (green), CD31 (red), and DAPI nuclear counterstain (blue) in the subventricular zone of the brain. Colocalized BrdU+/CD31+ proliferating endothelial cells appear yellow. Scale bar = 100 μm. (B) Quantitative analysis of the number of BrdU+/CD31+ double positive cells per field of view. (C) Network diagram of hub genes involved in angiogenesis associated with G‐3702 treatment in ischemic stroke. (D) Quantitative PCR analysis of mRNA expression of angiogenesis‐related genes BDNF, NAMPT, FGF2, ITGAV, ITGB3, SRC, RHOA, and VEGFA. *n* = 6 mice per group, presented as means ± SEMs. Significant differences are indicated by *p < 0.05, **p < 0.01, ***p < 0.001, and “NS” denotes no significant difference.

Immunofluorescence staining of CD31 was used to quantify vessels, while dual labeling of BrdU and CD31 identified proliferating endothelial cells. The Figure 8A show the number of BrdU/CD31 double positive cells in the peri‐infarct cortex across treatment groups as assessed by immunofluorescence. While the M group exhibited a statistically significant 1.72‐fold increase in double positive cells compared to NC, indicating some activation of angiogenesis following stroke, the extent was limited.

G‐3702 treatment groups showed a dose‐dependent enhancement of newly formed blood vessels marked by increased double labeling. Specifically, the LD, MD, and HD groups exhibited fold changes of 1.76, 4.98, and 3.12, respectively relative to NC, representing substantially greater proangiogenic effects compared to M. Among the G‐3702 doses, MD yielded the highest number of double positive cells. This confirms preferential promotion of angiogenesis downstream of ischemia‐induced pathways represents a key aspect of G‐3702's therapeutic mechanism of action in IS.

Based on metabolomics of G‐3702 in ischemic mice, the Avβ3 integrin pathway and PI3K‐Akt signaling pathway were implicated. These intersect in angiogenesis regulation. Pathway analysis of angiogenesis‐related genes in GSE58294 identified BDNF, NAMPT, FGF2, ITGAV, ITGB3, SRC, RHOA, and VEGFA as core angiogenesis genes (Figure 8C).

To validate these predictions experimentally, we performed qPCR on angiogenesis‐related targets (Figure 8D). In the M group versus NC, BDNF, FGF2, ITGAV, ITGB3, SRC, and RHOA mRNA were significantly downregulated (*p* < 0.05), indicating impaired angiogenesis [50, 51, 52, 53]. Treatment with G‐3702 dose‐dependently increased the mRNA of these genes versus M, with MD being highest (*p* < 0.05).

NAMPT mRNA rose in the M group versus NC, suggesting compensatory upregulation (*p* < 0.05) [54]. G‐3702 MD significantly reduced NAMPT mRNA (*p* < 0.05), potentially modulating endothelial function. VEGFA showed a nonsignificant increase in the M group versus NC, insufficient for repair [55]. G‐3702 stimulated a nonsignificant elevation, enhancing angiogenic regulation. Notably, VEGFA promotes angiogenesis through endothelial proliferation/permeability. Upregulation in the M group reflects compensatory cerebrovascular responses. BDNF enhances neurogenesis, white matter recovery, and angiogenesis poststroke. NAMPT exerts neuroprotective effects through NAD activation and proliferation/tone modulation of endothelial cells. SRC, FGF2, and RHOA regulate angiogenesis under various stimuli, including VEGFA and permeability. Their downregulation in the M group versus NC implicates impaired angiogenic signaling, explaining the therapeutic effects of G‐3702.

These results validate that modulation of proangiogenic pathways, particularly VEGFA, BDNF, and NAMPT, underlies G‐3702's mechanism in stroke recovery.

## 4 Conclusion

This study aimed to provide a more holistic understanding of IS pathogenesis by integrating metabolomic profiling with upstream pathway analysis and machine learning approaches. Untargeted metabolomic profiling of stroke mice revealed significant alterations in metabolic pathways that were effectively modulated by G‐3702 treatment, most notably the restoration of taurine and hypotaurine metabolism crucial for cellular homeostasis. By mapping how these metabolic changes corresponded to transcriptional variations in relevant disease datasets, insights were gained into the molecular drivers of stroke onset and progression implicated in the neuroprotective effects of G‐3702.

Network analysis further highlighted pathways modulated by G‐3702 in a dose‐dependent manner, most significantly by the medium dose. This dose achieved the greatest restoration of metabolic balance and the most significant mitigation of motor impairments and neuronal damage induced by stroke. While the medium dose demonstrated the most pronounced therapeutic effects, further studies are required to fully assess its safety profile, including potential hepatotoxicity and other adverse effects.

Insights were also provided into G‐3702's effects on angiogenesis‐related pathways such as integrin signaling and the PI3K/AKT pathway. Specifically, the results indicated G‐3702 may facilitate recovery poststroke by promoting angiogenesis and enhancing vascular remodeling through the Avb3 integrin pathway.

In summary, this integrated metabolomics‐transcriptomics approach enhanced the understanding of G‐3702's multifaceted neuroprotective mechanisms of action in IS. The study, based on a well‐established mouse model of IS, revealed that G‐3702 modulates key pathways involved in angiogenesis, inflammation, and cell survival, all of which are crucial for neuroprotection and stroke recovery. The results highlight the therapeutic potential of G‐3702, particularly at the optimal medium dosage, which showed the most pronounced effects in promoting recovery and reducing tissue damage. This targeted approach, aimed at modulating specific molecular pathways, offers a promising therapeutic avenue for stroke rehabilitation and warrants further investigation through preclinical and clinical studies to validate its efficacy and safety for human use.

## Author Contributions

C.W., Q.Z., and F.Z. conducted the experiments. C.W. and Q.Z. analyzed the data. C.W. and F.Z. wrote the main manuscript text. C.W. and J.W. prepared all figures. All authors reviewed and approved the manuscript.

## Ethics Statement

All experimental procedures were conducted in accordance with the guidelines and regulations approved by the Institutional Animal Care and Use Committee at Nanjing University of Science and Technology (Approval ID: ACUC‐NUST‐20230609).

## Consent

All participants provided informed consent.

## Conflicts of Interest

The authors declare no conflicts of interest.

## Data Availability

The original contributions presented in the study are included in the article. Further inquiries can be directed to the corresponding authors.
